# High-content screening image dataset and quantitative image analysis of *Salmonella* infected human cells

**DOI:** 10.1186/s13104-019-4844-5

**Published:** 2019-12-16

**Authors:** Antony N. Antoniou, Simon J. Powis, Janos Kriston-Vizi

**Affiliations:** 1 The Advanced Centre for Biochemical Engineering, University College London, Gower Street, London, WC1E 7JE UK; 2 Division of Infection and Immunity/Centre of Rheumatology, University College London, Rayne Building, 5 University Street, London, WC1E 6JF UK; 3 Faculty of Life and Health Sciences, Northumbria University, Ellison Building, Tyne and Wear Newcastle upon Tyne, NE1 8ST UK; 4 School of Medicine and Biological Sciences Research Complex, University of St. Andrews, St. Andrews, Fife KY16 9TF Scotland; 5 Laboratory for Molecular Cell Biology, Medical Research Council, University College London, Gower Street, London, WC1E 6BT UK

**Keywords:** *Salmonella*, Unfolded protein response, Endoplasmic reticulum, High-content screening, Image-based screening, Phenotypic screening, Confocal image, Cellular morphology, HeLa

## Abstract

**Objectives:**

*Salmonella* bacteria can induce the unfolded protein response, a cellular stress response to misfolding proteins within the endoplasmic reticulum. *Salmonella* can exploit the host unfolded protein response leading to enhanced bacterial replication which was in part mediated by the induction and/or enhanced endo-reticular membrane synthesis. We therefore wanted to establish a quantitative confocal imaging assay to measure endo-reticular membrane expansion following *Salmonella* infections of host cells.

**Data description:**

High-content screening confocal fluorescence microscopic image set of *Salmonella* infected HeLa cells is presented. The images were collected with a PerkinElmer Opera LX high-content screening system in seven 96-well plates, 50 field-of-views and DAPI, endoplasmic reticulum tracker channels and *Salmonella* mCherry protein in each well. Totally 93,300 confocal fluorescence microscopic images were published in this dataset. An *ImageJ* high-content image analysis workflow was used to extract features. Cells were classified as infected and non-infected, the mean intensity of endoplasmic reticulum tracker under *Salmonella* bacteria was calculated. Statistical analysis was performed by an *R* script, quantifying infected and non-infected cells for wild-type and Δ*sifA* mutant cells. The dataset can be further used by researchers working with big data of endoplasmic reticulum fluorescence microscopic images, *Salmonella* bacterial infection images and human cancer cells.

## Objective

*Salmonella* bacterial infections can lead to the development of inflammatory arthritis, known as Reactive Arthritis (ReA) within a subgroup of patients predominantly expressing the Human Leukocyte Antigen (HLA) class I molecule HLA-B27 [1, 2]. ReA is a member of the inflammatory arthritic diseases known as the spondyloarthropathies, which have been proposed to arise from the induction of the unfolded protein response (UPR), a cellular stress response to misfolding proteins within the endoplasmic reticulum (ER). HLA-B27 has been proposed to contribute and/or initiate the UPR [3]. The expression of HLA-B27 can contribute to enhanced *Salmonella* recovery [4]. We therefore proposed that *Salmonella* could exploit the UPR environment and induce the UPR. Recently, we have established that following *Salmonella* infection of host epithelial cells, the unfolded protein response (UPR) is activated. *Salmonella* exploit the UPR response to enhance bacterial replication, partly through UPR induced lipid biosynthetic pathways [5]. Previously, it has been established that part of the UPR pathway leads to the expansion of ER membranes through the activation and/or regulation of lipid biosynthetic pathways [6]. Therefore, we wanted to establish a confocal imaging method that could quantitatively determine endo-reticular membrane expansion, across a range of *Salmonella* infection conditions.

The quantification of endo-reticular membrane content was needed in infected cells in order to assess the increase of endo-reticular membranes due to *Salmonella* infection. ER tracker staining was used for the quantification of endo-reticular membrane content in infected cells.

The image dataset, ImageJ [7] macro and R [8] script presented here can be useful not only for the molecular biologist and biomedical researchers focusing to *Salmonella* providing them with an open-source software-based data analysis pipeline, but to the wider bioimage analysis community. Thousands of high-quality fluorescence nuclear, ER and *Salmonella* images can be used by software developers of image processing algorithms.

## Data description

The data report here (Table 1) is a high-content screening confocal fluorescence microscopic image set of *Salmonella* infected HeLa cells and its analysis.

**Table 1:** Overview of data files

Label	Name of data file/data set	File types (file extension)	Data repository and identifier (DOI or accession number)
Plate [1–7] _365nm.zip_	DAPI channel, 365 nm excitation wavelength, 16 bit pixel depth confocal fluorescence microscopic images, stacks of 96 well plates (7 files)	Image stacks saved in LZW compressed, native ImageJ .zip format that can be opened with ImageJ after renaming the extension from .zip_ to .zip	Harvard Dataverse [10]
Plate [1–7] _488nm.zip_	ER tracker channel, 488 nm laser excitation wavelength, 16 bit pixel depth confocal fluorescence microscopic images, stacks of 96 well plates (7 files)	Image stacks saved in LZW compressed, native ImageJ .zip format that can be opened with ImageJ after renaming the extension from .zip_ to .zip	Harvard Dataverse 10.7910/DVN/FYGHFO
Plate [1–7] _561nm.zip_	mCherry *Salmonella* channel, 561 nm laser excitation wavelength, 16 bit pixel depth confocal fluorescence microscopic images, stacks of 96 well plates (7 files)	Image stacks saved in LZW compressed, native ImageJ .zip format that can be opened with ImageJ after renaming the extension from .zip_ to .zip	Harvard Dataverse 10.7910/DVN/FYGHFO
ST_exp60_confocal_infection_plates.xls	Plate layout including ERT concentrations	MS Excel file (.xls)	Harvard Dataverse 10.7910/DVN/FYGHFO
Plate 2_Thr = 235.tif.zip_	Segmented 8 bit pixel depth binary images, stacks of 96 well plates	Image stacks saved in LZW compressed, native ImageJ .zip format that can be opened with ImageJ after renaming the extension from .zip_ to .zip	Harvard Dataverse 10.7910/DVN/FYGHFO
Plate 3_Thr = 180.tif.zip_	Same as above	Same as above	Same as above
Plate 4_Thr228-7275.tif.zip_	Same as above	Same as above	Same as above
Plate 5_Thr = 270.tif.zip_	Same as above	Same as above	Same as above
Plate 6_Thr = 197_sizefiltered_250px- = 26um2-.tif.zip_	Same as above	Same as above	Same as above
Plate 7_Thr = 177_sizefiltered_250px- = 26um2-.tif.zip_	Same as above	Same as above	Same as above
ImageJ_macro_ijm.txt	ImageJ macro file	Text file (.txt)	Harvard Dataverse 10.7910/DVN/FYGHFO
Infected_cell_ER_signal_Plate 2–7.zip_	Table containing extracted features as a result of image analysis	LZW compressed (.zip) Comma Separated Value (.csv) text files that can be opened for further processing with R or MS Excel after renaming the extension from .zip_ to .zip and uncompress	Harvard Dataverse 10.7910/DVN/FYGHFO
Plates 2–3.R	R script	Text file (.txt)	Harvard Dataverse 10.7910/DVN/FYGHFO
Plates 4–5.R	R script	Text file (.txt)	Harvard Dataverse 10.7910/DVN/FYGHFO
Plates 6–7.R	R script	Text file (.txt)	Harvard Dataverse 10.7910/DVN/FYGHFO

### Plate layouts

Plate 1: A non-infected control plate was used, which contained HeLa cells were not infected with *Salmonella enterica* and were stained with varying endoplasmic reticulum (ER) tracker (ERT) concentrations.

Plates 2–7: HeLa cells were infected with either wild-type *Salmonella enterica* or the isogenic *S. enterica Typhimurium* Δ*sifA* mutant using various multiplicity of infections (MOI) and were fixed 4, 16 or 24 h post infection.

### Image acquisition equipment and experimental setup

Confocal fluorescence microscopy images were acquired during a high-content screening session. Opera LX (PerkinElmer) confocal microscope was used for imaging (40 × air objective, NA = 0.6). Exposure times were used as follows: 100 ms for the DAPI-stained nuclear channel (365 nm excitation wavelength), 2000 ms for the ER tracker channel (488 nm laser excitation wavelength), 2000 ms for the *Salmonella* that constitutively expressed the mCherry fluorescent protein (561 nm laser excitation wavelength). 2 by 2 camera pixels were binned (integrated) resulting in a pixel size of 0.323  ×  0.323 μm. 50 field-of-view (FoV) images were acquired in each well, 4800 per 96-well plate.

### Image processing and data analysis

The image processing software was performed with ImageJ and the statistical data was analyzed with R.

The 561 nm channel image stacks were segmented using the highest pixel intensity of a given image stack as higher threshold value. The lower threshold was specified manually based on visual inspection in order to exclude the out of focus pixels. Size filter of 26 μm^2^ (250 pixel) was applied to plate 6 and 7 because of the presence of *Salmonella* Containing Vesicles containing large numbers of bacteria. The segmentation resulted in the binary mask of the *Salmonella* bacteria particles and the mean intensity of ER tracker pixels in 488 nm channel was measured under each cell. Cells were labeled as either “infected” or “non-infected” based on the presence or absence of *Salmonella* bacteria particles. Each cell with its fluorescence values can be correlated with its image based on their well position identifier (label column) in the extracted feature measurement file and the plate layout file.

The statistical data analysis was conducted by R scripts designed to process 4 h (plates 2–3), 16 h (plates 4–5) and 24 h (plates 6–7) post-infection together respectively and are available in the dataset of this paper. The workflow separated the intensity values infected and non-infected cells into separate files. This design provides the advantage that the high-throughput workflow can be done by a powerful R script, while flexibility is given to perform the significance test with any statistical application. Initially, the ImageJ macro-generated result files from multiple FoVs were opened. The script automatically opens all of the generated.csv files in a specific folder. Consecutively, the infected and non-infected cells for wild-type and Δ*sifA* mutant cells were identified and saved into separate text files respectively. That result was used for significance test, reported in Ref. [5].

## Limitations

Camera binning, integration of 2 by 2 pixels was used in order to maximize signal strength. That resulted in the fourfold increase of signal. However, the effective resolution of the microscope’s CCD camera was reduced accordingly to 671 × 497 pixels.

The described implementation of the image processing pipeline required a PC that is equipped with enough RAM memory (e.g. 32 GB) where a channel of a plate’s stack can be loaded and processed.

Infections were performed at 60–80% confluency and therefore cell density was not uniform in every FoVs. This limitation was addressed during image processing by analyzing FoVs with higher mean intensities in their nuclear channel.

The method has only been tested in a single cell line. The HeLa cell line was chosen on the basis that HeLa cells do not express Toll Like Receptor (TLR) ligands. HeLa cells along with other epithelial cell lines such as 293T were assay for TLR activation using a TLR-NF-kB reporter. HeLa cells demonstrated a lack of TLR expression. The reasoning behind using a TLR negative cell line is that it has been previously reported that TLR engagement can activate the UPR activated transcription factor XBP-1 [9] which can affect lipid and ER membrane biosynthesis. We therefore required conditions which would best dissect impact of *Salmonella* on the UPR and ER membrane synthesis, without additional TLR mediated effects. Therefore, for our analysis to be extended into further cell types, the potential contribution of innate receptor engagement to UPR induction and ER biosynthesis must be taken into account.

## Data Availability

The data described in this Data Note can be freely and openly accessed on Harvard Dataverse 10.7910/DVN/FYGHFO. Please see Table 1 and reference list for details and links to the data.

## Abbreviations

ER: endoplasmic reticulum
ERT: endoplasmic reticulum tracker
UPR: unfolded protein response
MOI: multiplicity of infections
OME: open microscopy environment
FoV: field-of-view
Thr: threshold
